# Topics of the Month

**Published:** 1897-03

**Authors:** 


### TOPICS OF THE MONTH.

Hon. William P. Letchworth, who for twenty-three years has been a member, and a portion of the time president, of the State Board of Charities, recently made an inspection of the poor-houses of the eighth judicial district. From this report we glean some interesting data. It appears that when this inspection was made there were 1,151 inmates in the eight poor-houses in the district. Of this number 69 per cent. were men and 31 per cent. women. The number of men has increased since 1869, while the number of women has decreased, as there was then 56 per cent. men and 44 per cent. women. The decrease in the number of women is attributed, in part, to the increased number of avenues open to their employment and to their more temperate habits, especially in the use of tobacco and alcohol.

The inmates in general are reasonably well fed and clothed, according to Mr. Letchworth, though in some instances undercloth-ing was needed. A greater variety of foods, especially vegetables, might be furnished by some of the institutions without increasing the expense and with general beneficial influence. There had been some improvement in the care of the sick; especially had there been greater attention paid to bathing and general personal cleanliness. Some of the poor-houses, however, were insufficiently supplied with water, a defect that ought to be speedily remedied.

He pointed out some defects in the Erie County poor-house which may be grouped under the general statement that there is lack of proper discipline, suitable care and healthful employment of the inmates, together with want of economical methods of purchasing supplies, and a deficiency in general good administration. The closets and storerooms are disordered, there is a want of systematic order on the premises, indicated by the improper disposition of waste and rubbish ; a neglect of the grounds, as seen in the growth of tall weeds about the yards and paths ; a limited garden space and unproductive farming.

Mr. Letchworth is an expert on the subject of which he writes, and while he admits that there has been a general improvement in the care of the poor during the past twenty years, he yet thinks that Erie county is considerably behind other counties in the conduct and efficiency of its almshouse. This report ought to stimulate improvement on the part of the officials who control this important charity.

---

KOCH'S mission to South Africa, upon which he set out about the middle of November last, is one of exceptional interest. It seems that the foremost living bacteriologist has been commissioned to discover and deal with the supposed germs of one of the most contagious and destructive of epizootic diseases. This malady is ravaging a large portion of the continent, impoverishing millions of people, besides working incalculable harm in many and divers ways not easily mentioned. This rinderpest, or cattle plague, is not a new disease, for it has been known for nearly two centuries. It has not appeared, however, in this country until late years. There was an outbreak of it in Great Britain in 1865, when the government ordered the slaughter of all infected herds. Little is known of its etiology, though when it raged in England in 1865 it was studied by a royal commission, with Sir Richard Quain at its head. That was, however, before bacteriology was known as a science, hence the inquiry resulted in little more than proving the contagiousness of the disease and the futility of the then known methods of treatment.

Dr. Koch will, of course, investigate it chiefly from the bacteriological standpoint, and he will be assisted by his colleague, Dr. Kohlstock, and perhaps others. We await with considerable interest the result of this investigation.

---

CHRISTIAN SCIENCE, so-called, a pretended method of cure that is neither Christian-like nor scientific, is having hard lines in Castile,

Wyoming county, N. Y. The services of a coroner were called for, who empaneled a jury to investigate the circumstances attending the death of a child which have caused indignation and excitement in that community. It appears, according to reports, that the services of a physician were dispensed with and that "Christian science" was invoked to conduct a labor. There is much indignation over the circumstances, and on Sunday, February 14th, the pastor of the Presbyterian church in that village delivered a sermon in which he severely denounced "Christian science" and those who practise it. He very justly claimed that it was opposed to Christianity and all the sciences, and affirmed that it was quite time for the people to take issue against such doctrine when murder had been committed in the village by the practice of "Christian science" in a case of confinement.

This reverend divine has distinguished himself by propounding most sensible conclusions, and it would be well if other clergymen followed his estimable example.

The coroner's jury has severely censured all connected with the unfortunate affair, especially one of the Christian science fiends from Buffalo as chiefly responsible.

---

UNDER the title Sanitary trivialities, the *Journal of the American Medical Association*, in its issue of January 30, 1897, makes editorial comment upon the ordinance passed by the common council of Buffalo relating to the sale of nursing bottles. The *Journal* very properly observes that the passage of the ordinance referred to signalised another victory for the commissioner of health, Dr. Ernest Wende, over popular incredulity and ridicule. Buffalo, says the *Journal*, has already accomplished a great deal in municipal sanitation. The regulation of the milk supply and the identification and isolation of diseased cattle in herds are all-sufficient. A register, too, is kept in the health office of the location of every fatal case of diphtheria, scarlet and typhoid fevers, and if these are found to occur along any special milk delivery route, all the milk on that route is carefully traced to the dairy, thence to the herd.

After making other general remarks, the *Journal* concludes as follows: "One is confronted everywhere by apparently trivial sanitary neglects, each of which in its possibilities for evil will be found anything but trifling. Dr. Wende deserves to be congratulated on his success in suppressing the rubber tube of the nursing bottle, and the city of Buffalo for having a commissioner of health who is not deterred by ridicule from attacking so-called sanitary trivialities."

---

THE season is again upon us when mischievous bills relating to education are flooding the legislature at Albany. Of the total number at present introduced bearing on this question we are not advised, but we are familiar with two, the pernicious effects of which, if passed, would be far-reaching. One of these has been introduced into both houses, and has for its object the incorporation of the so-called New York Law School, which is a private institution already chartered by the regents, but subject to their control as to preliminary requirements and rules for graduation. The bill presented seeks to confer upon the school authority to regulate its own standards.

The second bill to which we invite attention is known as the "optometry bill," introduced in the assembly by Mr. Horton, of Wayne, and is No. 459. This measure seeks to confer the right upon opticians to examine eyes with reference to refractive errors under the influence of mydriatics or otherwise, as is now only permitted to be practised by regularly educated licensed physicians. The effect of this bill, if passed, will be to degrade the high office of physician, and it would be otherwise pernicious in making inroads upon the present splendid educational system as administered by the regents of the University of the state of New York at Albany. Educators, physicians, scientists and public-spirited citizens should unite in opposing both of these measures, that are simply introduced in the interest of individuals and are opposed to the interests of the general public.

---

IT IS interesting to note that progress is making in reference to questions pertaining to public health in many portions of the country. In Michigan quarterly meetings are held in different parts of the state under the direction of the state board of health, in which the local health authorities meet for conference and discussion of sanitary problems. In Pennsylvania there is an organisation called the Associated Health Authorities, which has lately held its fourth annual meeting at Harrisburg. It was opened with an address by Governor Hastings, president of the organisation, who said, among other things, that he was interested in no subject more than in that of public health. Pennsylvania, he said, instead of being at the foot of the list of states as she is in all sanitary measures, should be in the lead ; that the time had come when she should have laws enacted for the preservation of the health of her people, for the collecting of vital statistics, the protection of her water supplies, and the furtherance of her sanitary work. He called attention to the fact that in the great state of Pennsylvania the present legislation covers the half of it and he hoped that these associated health authorities would remedy the condition. A number of interesting papers were read and discussed and the meeting adjourned after manifesting much enthusiasm.

